# Apocynin-Treatment Reverses Hyperoxaluria Induced Changes in NADPH Oxidase System Expression in Rat Kidneys: A Transcriptional Study

**DOI:** 10.1371/journal.pone.0047738

**Published:** 2012-10-16

**Authors:** Sunil Joshi, Benjamin T. Saylor, Wei Wang, Ammon B. Peck, Saeed R. Khan

**Affiliations:** 1 Department of Pathology, Immunology and Laboratory Medicine, University of Florida College of Medicine, Gainesville, Florida, United States of America; 2 Department of Oral Biology, University of Florida College of Dentistry, Gainesville, Florida, United States of America; 3 Department of Infectious Diseases and Pathology, University of Florida College of Veterinary Medicine, Gainesville, Florida, United States of America; Emory University, United States of America

## Abstract

**Purpose:**

We have previously shown that production of reactive oxygen species (ROS) is an important contributor to renal injury and inflammation following exposure to oxalate (Ox) or calcium-oxalate (CaOx) crystals. The present study was conducted, utilizing global transcriptome analyses, to determine the effect of Apocynin on changes in the NADPH oxidase system activated in kidneys of rats fed a diet leading to hyperoxaluria and CaOx crystal deposition.

**Approach:**

Age-, sex- and weight-matched rats were either fed regular rat chow or regular rat chow supplemented with 5% w/w hydroxy-L-proline (HLP). Half of the rats on the HLP diet were also placed on Apocynin-supplemented H_2_O. After 28 days, each rat was euthanized, their kidneys freshly explanted and dissected to obtain both cortex and medulla tissues. Total RNA was extracted from each tissue and subjected to genomic microarrays to obtain global transcriptome data. KEGG was used to identify gene clusters with differentially expressed genes. Immunohistochemistry was used to confirm protein expressions of selected genes.

**Results:**

Genes encoding both membrane- and cytosolic-NADPH oxidase complex-associated proteins, together with *p21rac* and *Rap1a*, were coordinately up-regulated significantly in both renal medulla and cortex tissues in the HLP-fed rats compared to normal healthy untreated controls. Activation of NADPH oxidase appears to occur via the angiotensin-II/angiotensin-II receptor-2 pathway, although the DAG-PKC pathway of neutrophils may also contribute. Immuno histochemical staining confirmed up-regulated gene expressions. Simultaneously, genes encoding ROS scavenger proteins were down-regulated. HLP-fed rats receiving Apocynin had a complete reversal in the differential-expression of the NADPH oxidase system genes, despite showing similar levels of hyperoxaluria.

**Conclusions:**

A strong up-regulation of an oxidative/respiratory burst involving the NADPH oxidase system, activated via the angiotensin-II and most likely the DAG-PKC pathways, occurs in kidneys of hyperoxaluric rats. Apocynin treatment reversed this activation without affecting the levels of hyperoxaluria.

## Introduction

Oxalate (Ox) is a naturally-occurring, highly oxidized organic compound with powerful chelating activity that can cause death at high concentrations in animals and occasionally humans due to its toxic corrosive effects on cells. More commonly, however, higher concentrations of Ox in human fluids can cause a variety of pathological disorders, including hyperoxaluria, cardiomyopathy, cardiac conductance disorders, renal failure and, in particular, calcium oxalate (CaOx) nephrolithiasis [1]–[3]. Although oxalate can be absorbed by all segments of the intestinal tract, the large intestine appears to be where greatly enhanced oxalate absorption occurs in patients with enteric hyperoxaluria due to ileal disease [4], [5], chronic inflammatory bowel disease [6], as well as fat malabsorption, steatorrhea and sprue [5]. Enteric hyperoxaluria is also a well-documented entity observed in gastrointestinal diseases, such as colitis or Crohn's disease or following ileal resection in jejuno-ileal bypass surgery, and now certain bariatric surgeries for obesity [6]–[10].

Although Ox is endogenously produced via liver metabolism, dietary Ox is also a major contributor to urinary Ox excretion in most individuals, with recent studies indicating that dietary Ox can contribute as much as 50% of the daily urinary oxalate excretion [7], [8]. Unfortunately, hyperoxaluria even in the absence of elevated calcium can induce CaOx crystallization in the kidneys, suggesting a prominent role for both urinary calcium and oxalate in the formation and retention of CaOx crystals in renal tissues of susceptible individuals and subsequent formation of kidney stones. Pathologically, evidence clearly points to activation of a number of pathways [11]–[13] and renal injury in the presence of either hyperoxaluria or calcium oxalate crystal depositions in renal tissue [14].

In order to study the dynamics of calcium oxalate nephrolithiasis, nephrocalcinosis, metabolic acidosis, hematuria and renal failure, a number of animal models have been developed. The best studied models involve the ingestion of ethylene glycol (EG) or hydroxy-L-proline (HLP). Rats treated with EG or HLP develop hyperoxaluria leading to crystal deposition and production of reactive oxygen species (ROS), lipid peroxidation and cellular injury [15]–[21]. ROS induce up-regulated expression of various factors, including bikunin, osteopontin, Tamm-Horsfall protein, and matrix Gla protein, each thought to play a protective role against renal injury [14], [22]–[24].

**Figure 1:**
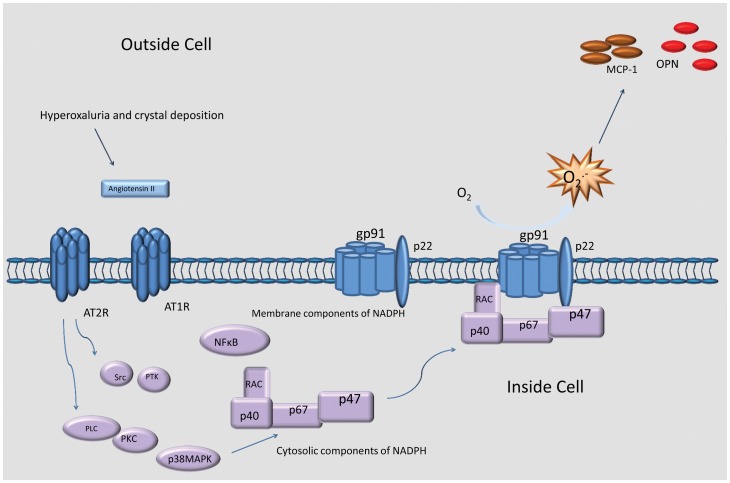
Summary of the proposed activation of Nox-2 and Nox-4 NADPH oxidases in the kidney. The figure shows various cytosolic and membrane components of NADPH oxidase subunits getting activated via Renin/angiotensin receptor-2 activation of the NADPH-oxidases during the development of hyperoxaluria, resulting in increased production of reactive oxygen species (ROS).

Earlier studies from our laboratory [23]–[26], as well as others [27]–[31], [34], have shown that cellular exposure to Ox and/or CaOx crystals leads to activation of NADPH oxidase, production of ROS and increased expression of molecules such as osteopontin (OPN) and monocyte chemoattractant protein-1 (MCP-1/Ccl2). These changes could be reduced by NADPH oxidase inhibitors, e.g., DPI [25]–[29] and Apocynin [31]. Although ROS may be produced in numerous non-phagocytic cell types by either the NADPH oxidase complex or an NADH-dependent ROS-generating activity, the respiratory burst of phagocytes of the innate immune system, in particular neutrophils and macrophages, is a result of NADPH oxidase activation [35]. NADPH oxidase is a dynamic complex comprised of a membrane-associated low potential heterodimeric flavocytochrome b_558_ plus several cytosolic components that translocate to the membrane cytochrome. Flavocytochrome b_558_ consists of three subunits, gp91*^Phox^* (also referred to as Nox2), p22*^Phox^* and Rap1a. While the flavocytochrome contains all the catalytic machinery required for electron transfer from NADPH to molecular O_2_, regulated activation of NADPH oxidase requires the translocation of several cytosolic regulatory subunits, p47*^Phox^*, p40*^Phox^* and p67*^Phox^*, plus the small G-protein p21rac, to the membrane where they associate with flavocytochrome b_558_. In the distal tubular cells of the renal cortex, the protein Nox4 substitutes for gp91*^Phox^* in the NADPH oxidase complex [36]. Irrespective, the activation of the NADPH oxidase pathway(s), which we predict are angiotensin receptor (Agtr)-activated in renal tissues, is depicted in Figure 1, and is the focus of the present study.

In the present study, we have analyzed changes in the global transcriptome of renal tissues following development of hyperoxaluria in rats fed HLP over a 4 week period, either with or without Apocynin treatment. All rats fed HLP developed hyperoxaluria as well as depositions of CaOx crystals in the kidneys. Results presented herein show an increase in the expression of genes involved in the activation of NADPH oxidase with a concomitant decrease in the expression of genes encoding the ROS scavenger proteins, catalase (Cat) and superoxide dismutase-1 (Sod1), in response to hyperoxaluria and crystal deposition. Oral treatment with Apocynin, a known inhibitor of NADPH oxidase complex assembly and oxidative stress, reversed not only the transcriptome profile of the NADPH oxidase-associated genes, but also multiple molecular pathways involving numerous cell components. These data raise the possibility that apocynin is a broad-spectrum anti-oxidant.

## Materials and Methods

### Animal model

Male Sprague–Dawley rats (n = 18), 8 weeks of age and weighing on average about 150 gm, were purchased from Harlan Laboratories and permitted to acclimate for 2 weeks within the University of Florida's Animal Care Facilities prior to any experimental procedures. Rats were divided into three groups of 6 each: Group 1 rats were fed a normal rat chow and sterile water, Group 2 rats received a diet similar to Group 1 rats, except the food was supplemented with HLP (ICN Biochemicals, Aurora, OH) to 5%, while Group 3 rats received a diet similar to Group 2 rats, except their water was supplemented with Apocynin to 4 mM. To prepare the rat chow, feed pellets were ground, then moistened with water and formed into “cookies”, which were individually weighed. All rats had free access to food and water, and consumption of food and intake of water were recorded daily. Each rat was weighed weekly to check its growth. At day 28 post-treatment, the rats were sedated, then euthanized and their kidneys freshly explanted. All procedures were approved by the University of Florida's IACUC and were in accord with recommendations of the NIH Guide for the Care and Use of Laboratory Animals.

### RNA preparation and detection of differentially expressed genes by microarray analyses

Preparation of total RNA from each of the 36 individual specimens was performed as described in detail elsewhere [25]. In brief, renal tissues were freshly excised after 4 weeks of HLP treatment, surgically separated into medulla and cortex, then snap-frozen in liquid nitrogen and stored at −80°C. Total RNA from a small piece of each cortex and each medulla from each rat within the three different treatment groups were isolated concurrently (for a total of 6 RNA preparations for each experimental group) using the RNeasy Mini-Kit (Qiagen, Valencia, CA), as per the manufacturers protocol, and single hybridizations were carried out with each of the 36 individual RNA samples using the IlluminaTM RatRef-12 Expression BeadChip, per manufacturer's instructions. This permitted a replicate of n = 6 for each comparative group from which to calculate a mean value and standard deviation. The RatRef-12 Expression BeadChip contained >22,000 genes expressed in the rat genome. All microarray data have been deposited with Gene Expression Omnibus (GSE36446).

### Microarray analysis and Data Mining

Microarray analysis was conducted at the University of Florida's Interdisciplinary Center for Biotechnology Research using the Illumina^TM^ bead array reader, while all gene expression data were analyzed using The Genome Studio Gene Expression Module V1.0. Before comparative analysis, the individual signal intensity values obtained from the microarray probes were log transformed (using 2 as the base) and normalized between all individual samples within the six sets in this study. After normalizing the signal intensity values for each of the 36 arrays, the Student's t-test was performed considering a probe-by-probe comparison between two groups at a time. In each comparison, p-value and fold changes (FC) were computed for each gene based on the n = 6 replicate samples within each experimental group and volcano plots were drawn for each comparison (Fig. S1). The analyses included gene expressions between cortex and medulla tissues from control versus HLP-treated rats and between control and HLP+Apocynin-treated rats. GO: TERM and KEGG pathway analyses based on differentially-expressed genes were carried out using DAVID (Database for Annotation, Visualization of Integrated Discovery) enrichment analysis tool from National Institute of Allergy and Infectious Diseases (NIAID), NIH [32]–[33]. Cluster analyses of genes permitted identification of biological processes, cellular component, and molecular function ontology.

### Histological examinations

Kidneys from each rat were surgically removed at time of euthanasia, one of which was used for RNA preparation, while the second was placed in 10% phosphate buffered formalin for 24 hours for eventual histological examinations. The formalin-fixed tissues were embedded in paraffin and sectioned to a thickness of 5-μm. Deparaffinization of paraffin-embedded slides was performed by xylene immersion and subsequent dehydration in ethanol. Slides were incubated overnight at 4°C with primary antibodies reactive to either Opn, MCP-1/Ccl2, p47 or Nox-2 (Abcam, Cambridge, MA). Isotype controls were performed using rabbit IgG. Slides were incubated for 30 min in biotinylated goat anti-rabbit IgG followed by incubation with biotinylated horseradish peroxidase using the Vectastain® ABC kit. Staining was developed by addition of diaminobenzidine (DAB) substrate (Vector Labs, Burlingame, CA) and counterstained with hematoxylin. To ensure that positive infiltrate staining for these antibodies was not due to high background staining, an additional run was performed using 10 mM citrate buffer for antigen-retrieval with all other procedures unchanged. Antigen-retrieval was carried out in 25 mMTris/EDTA buffer, pH 9.1 at 60°C for 20 min under 18 psi pressure. Images were taken using the Zeiss Axiovert 200M microscope (Carl Zeiss Microimaging, Inc., Thornwood, NY).

## Results

### Histological findings

**Figure 2:**
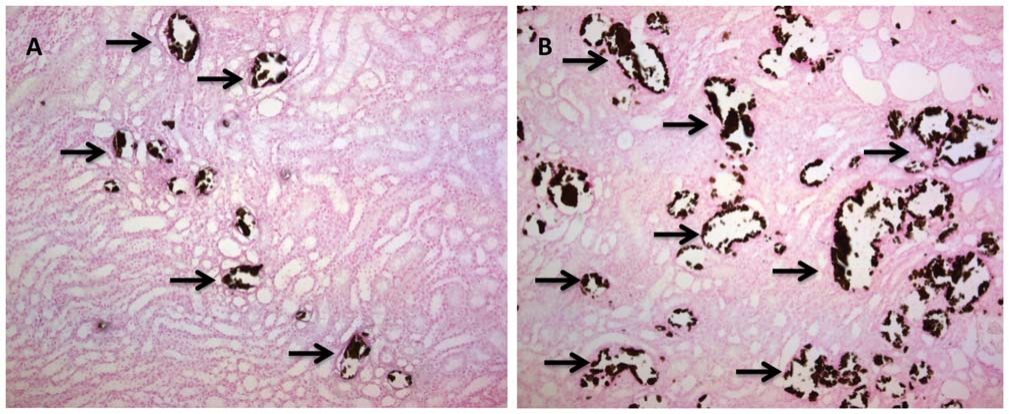
Representative Pizzolato staining of paraffin embedded kidney sections of Hydroxy-L-Proline (HLP) treated rats. (**A**) Light micrograph section showing little calcium oxalate crystal depositions shown with single arrows at 10X. (**B**) Light micrograph section of the kidney showing brownish-black stained calcium oxalate crystal depositions shown with single arrows with the oral administration of 5% hydroxy-l-proline (10X). Majority of the crystals were seen in the tubular lumens of the distal tubules and collecting ducts of the cortex and outer medulla. There were no crystal deposits in normal rat kidneys.

As anticipated, rats fed a HLP-free diet remained normo-oxaluric and devoid of any crystals, while the HLP-supplemented diet led to hyperoxaluria and variable levels of CaOx crystal depositions in all rats [31]. Kidney sections from HLP-fed rats revealed variable quantities of crystals (Fig. 2). Apocynin treatment led to some reduction in crystal deposition. Although crystals were present in all segments of the kidneys, including cortex, medulla and papilla, the majority were seen in the tubular lumens of the distal tubules and collecting ducts of the cortex and outer medulla. Smaller numbers could be found in the inner medulla and proximal tubules. The tubules that contained crystals were dilated and showed destruction of the lining epithelium as previously described [15]. The neighboring tubules appeared normal with no overt signs of injury.

### Genes associated with the Agtr-NADPH oxidase pathway are coordinately up-regulated in renal cortex and medulla tissues following HLP-treatment

Of the 21,000 rat genes represented on the array, 3302 and 2894 were found to be significantly differentially-expressed at a 2-fold change in the cortex and medulla, respectively, in a comparison between control and HLP-fed rats, while 3000 and 2505 genes, respectively, were found to be significantly expressed in the cortex and medulla in the comparison between control and HLP+Apocynin-fed rats. Using KEGG to curate the differentially-expressed genes, several gene clusters were identified that defined specific biological processes for the HLP-fed and HLP+Apocynin-fed rats, as presented in Tables 1 and 2 for cortex and medulla, respectively. Interestingly, the differentially-expressed genes of the cortex and medulla tissues identifying the various KEGG signaling pathways revealed that, while 10 pathways were shared, each tissue differed at 25 additional pathways. These data strongly indicate that the effects of both HLP and Apocynin on renal tissues are complex and involve distinct activities on cellular functions.

**Table 1:** Functional annotation chart showing differentially-expressed pathways in renal cortex tissue comparing HLP+Apocynin treated versus untreated control groups.

Category	Signaling Pathway	Count	Percentage	p-value	Benjamini
**KEGG_PATHWAY**	MAPK signaling pathway	29	3.5	3.6E-3	1.4E-1
**KEGG_PATHWAY**	TGF-beta signaling pathway	13	1.6	6.3E-3	1.9E-1
**KEGG_PATHWAY**	Fc epsilon RI signaling pathway	12	1.5	6.6E-3	1.7E-1
**KEGG_PATHWAY**	Chemokine signaling pathway	19	2.3	1.8E-2	2.1E-1
**KEGG_PATHWAY**	Gap junction	11	1.3	2.7E-2	2.3E-1
**KEGG_PATHWAY**	Complement and coagulation cascades	10	1.2	2.8E-2	2.2E-1
**KEGG_PATHWAY**	Focal adhesion	19	2.3	5.6E-2	3.5E-1
**KEGG_PATHWAY**	Insulin signaling pathway	14	1.7	6.4E-2	3.5E-1
**KEGG_PATHWAY**	ErbB signaling pathway	10	1.2	7.8E-2	3.9E-1
**KEGG_PATHWAY**	Non-small cell lung cancer	7	0.8	1.0E-1	4.1E-1
**KEGG_PATHWAY**	Steroid hormone biosynthesis	12	1.5	4.2E-5	6.8E-3
**KEGG_PATHWAY**	Cytokine-cytokine receptor interaction	25	3.0	9.9E-4	7.8E-2
**KEGG_PATHWAY**	Metabolism of xenobiotics by cytochrome P450	11	1.3	3.4E-3	1.7E-1
**KEGG_PATHWAY**	Jak-STAT signaling pathway	17	2.1	1.1E-2	2.4E-1
**KEGG_PATHWAY**	Autoimmune thyroid disease	10	1.2	1.2E-2	2.2E-1
**KEGG_PATHWAY**	Graft-versus host disease	9	1.1	1.2E-2	2.0E-1
**KEGG_PATHWAY**	GnRH signaling pathway	13	1.6	1.3E-2	1.9E-1
**KEGG_PATHWAY**	Allograft rejection	9	1.1	1.5E-2	2.0E-1
**KEGG_PATHWAY**	Arginine and proline metabolism	9	1.1	1.5E-2	2.0E-1
**KEGG_PATHWAY**	Calcium signaling pathway	20	2.4	1.7E-2	2.1E-1
**KEGG_PATHWAY**	Neuroactive ligand-receptor interaction	26	3.1	1.9E-2	2.0E-1
**KEGG_PATHWAY**	Taurine and hypotaurine metabolism	4	0.5	2.0E-2	2.0E-1
**KEGG_PATHWAY**	Long-term depression	10	1.2	2.1E-2	2.0E-1
**KEGG_PATHWAY**	Retinol metabolism	9	1.1	2.8E-2	2.2E-1
**KEGG_PATHWAY**	Drug metabolism	10	1.2	3.2E-2	2.4E-1
**KEGG_PATHWAY**	Antigen processing and presentation	11	1.3	4.4E-2	3.0E-1
**KEGG_PATHWAY**	Biosynthesis of unsaturated fatty acids	5	0.6	5.7E-2	3.4E-1
**KEGG_PATHWAY**	ECM-receptor interaction	10	1.2	6.1E-2	3.5E-1
**KEGG_PATHWAY**	Type I diabetes mellitus	8	1.0	7.5E-2	3.9E-1
**KEGG_PATHWAY**	Alpha-Linolenic acid metabolism	4	0.5	8.4E-2	4.0E-1
**KEGG_PATHWAY**	VEGF signaling pathway	9	1.1	8.5E-2	4.0E-1
**KEGG_PATHWAY**	Glutathione metabolism	7	0.8	8.6E-2	3.9E-1
**KEGG_PATHWAY**	Viral myocarditis	10	1.2	8.8E-2	3.8E-1
**KEGG_PATHWAY**	Purine metabolism	15	1.8	9.4E-2	4.0E-1
**KEGG_PATHWAY**	Hedgehog signaling pathway	7	0.8	1.0E-1	4.1E-1

The gene heading indicates number of genes mapped to an ontology category. The first ten pathways are common between the cortex and the medulla. P-values derived from Fisher's exact test and Benjamini multiple test correlation.

**Figure 3:**
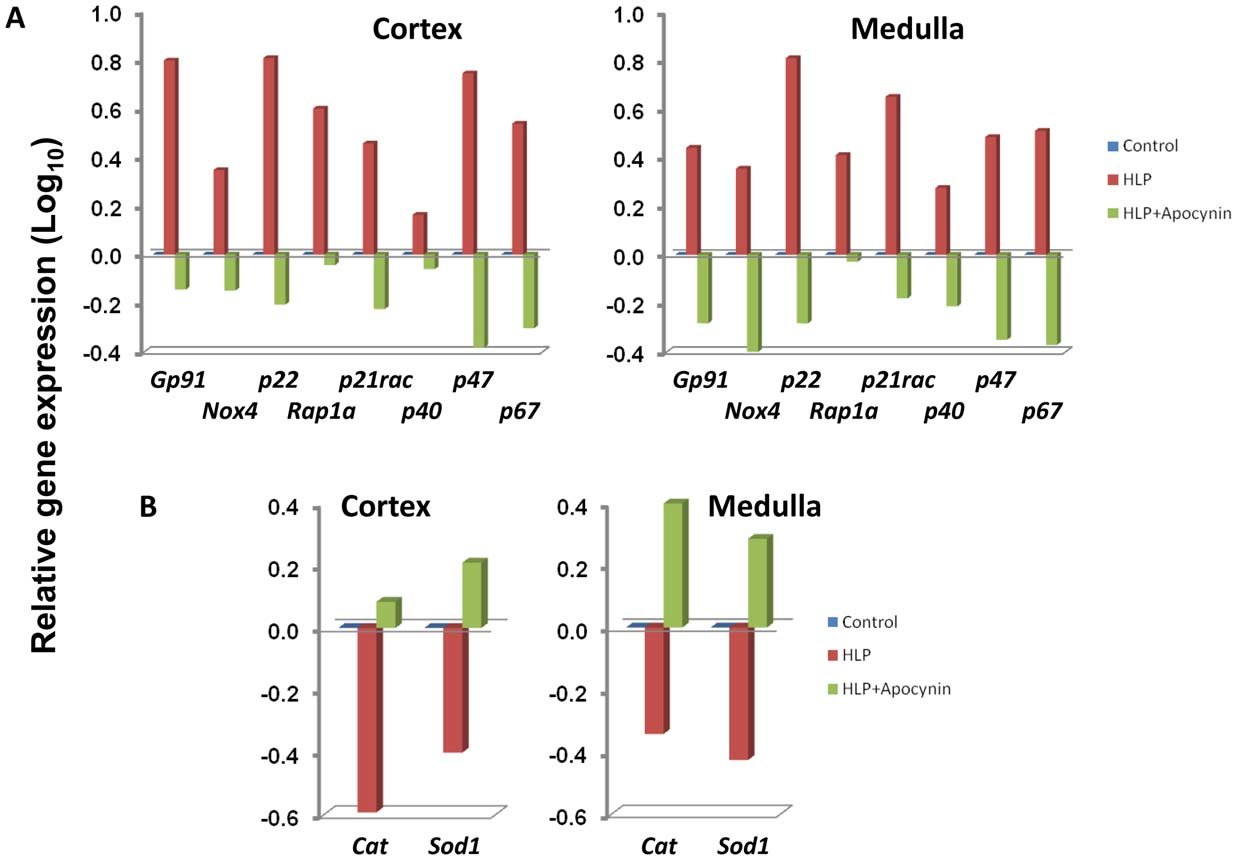
Relative expressions of genes in HLP-fed rats vs. control compared to HLP+ Apocynin fed rats vs. control. (**A**) Genes encoding the membrane components of NADPH oxidase such as gp91^Phox^ or Nox2, p22^Phox^, and Rap1a as well as cytosolic components such as p47, p40 and p67. gp91^Phox^ is substituted by Nox4 in the distal tubular cells of the renal cortex of the NADPH oxidase complex (**B**) Genes encoding the oxygen scavengers such as catalase (cat) and superoxide dismutase (sod1) are shown to be down regulated in the HLP-fed rats (Red bars) and up regulated in the HLP-Apocynin fed rats (Green bars). Most of the genes depicted had values >0.3 (or >2 fold ratio differential-expression).

Focusing specifically on the proteins involved in the activation of the Agtr-NADPH oxidase pathway *per se*, both cortex and medulla renal tissues exhibited similar differential gene expressions between non-treated controls versus HLP-treated rats. This included the genes encoding for the three subunits comprising both the phagocytic and renal epithelium Flavocytochrome b_558_ (*p22^Phox^*, *Rap1a and gp91^Phox^* or *Nox4*), the three cytosolic subunits (*p47^Phox^*, *p67^Phox^* and *p40^Phox^*), plus the small Rho-γ activated G-protein (*p21rac*). In contrast, genes encoding the oxygen radical scavengers *Sod1* (encoding superoxide dismutase-1) and *Cat* (encoding catalase) were down-regulated (Fig. 3). Interestingly, examination of the same gene profiles generated by a comparison between non-treated controls and HLP+Apocynin-treated rats revealed a complete reversal in gene expressions (Fig. 3).

**Figure 4:**
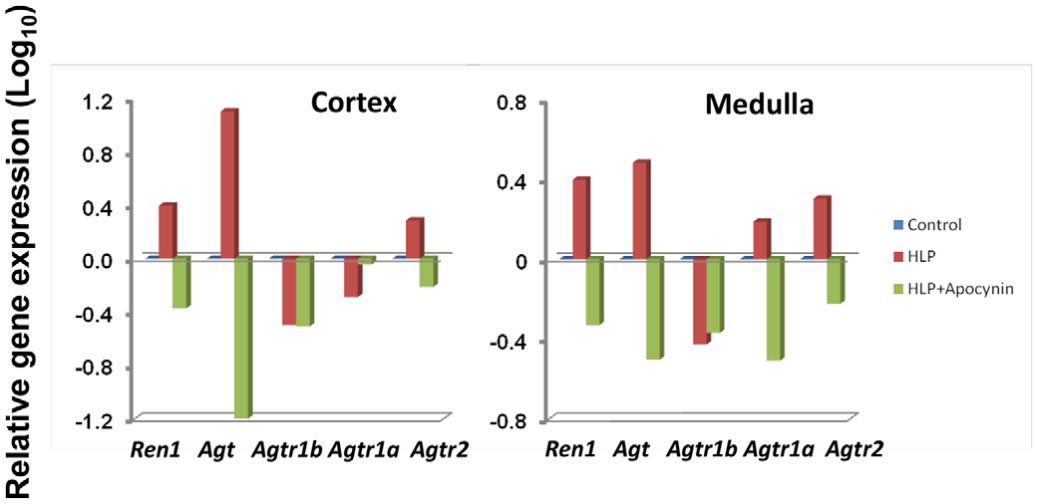
Relative expressions of genes in HLP-fed rats vs. control compared to HLP+ Apocynin fed rats vs. control. Genes encoding proteins of renin, angiotensin, and angiotensin receptor 2 were up regulated in the cortex and medulla of HLP-treated rats (Red bars) but interestingly down regulated in the HLP+Apocynin rats (Green bars). Angiotensin receptor 1and 1a showed differential expression for both the cortex and medulla in the HLP fed and HLP-Apocynin fed rats.

**Figure 5:**
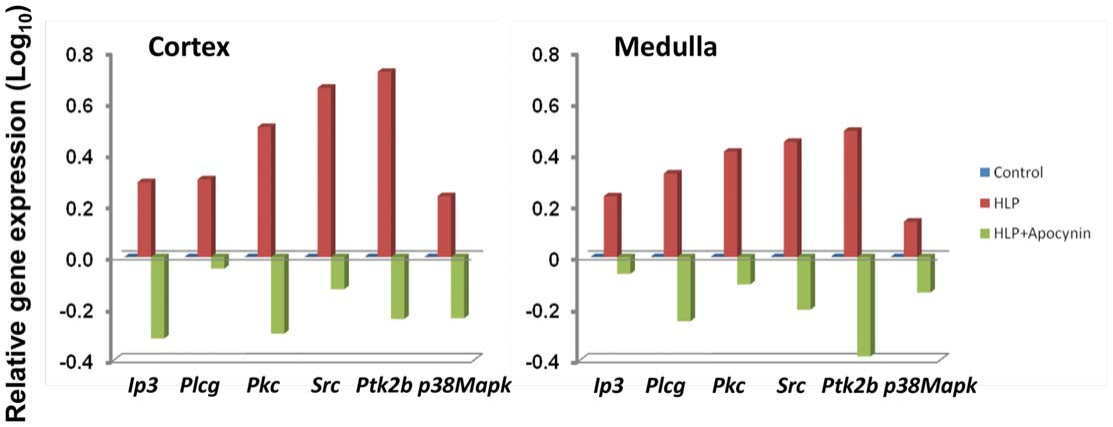
Relative expressions of genes in HLP-fed rats vs. control compared to HLP+ Apocynin fed rats vs. control. Genes downstream of angiotensin receptor-2 encoding important immune cell-associated signaling pathways for the activation of NADPH oxidase expressed in the cortex or medulla of HLP-treated rats showing up regulation (Red bars), but exhibiting down-regulation in the cortex and medulla of HLP+Apocynin-treated rats (Green bars).

In an attempt to define a possible mechanism activating the NADPH oxidase systems, we examined whether the rennin/angiotensin/angiotensin-receptor system might be involved. While the gene encoding angiotensin, *Agt, per se* was strongly up-regulated in the HLP-treated rats, the genes encoding the two subunits that comprise angiotensin-receptor-1 (*Agtr1a* and *Agtr1b*) were either unchanged or actually down-regulated (Fig. 4). In contrast, the gene encoding the homodimeric angiotensin-receptor-2 (*Agtr2*) was up-regulated; suggesting that one activation pathway for NADPH oxidase in the current system is directly through the Agtr2 signaling pathway. Again, this gene profile was reversed in the HLP+Apocynin-treated rats. Lastly, examination of various genes downstream of angiotensin-receptor-2, specifically *Ip3, Plcγ, Pkc, Src, Ptk2b*, and *p38Mapk*, revealed that each was up-regulated in both cortex and medulla tissues of HLP-fed rats, with *Ip3, Pkc, Src, Ptk2* and *p38Mapk* all down-regulated in HLP+Apocynin-fed rats (Fig. 5), suggesting that activation of NADPH oxidase is via both Src/Ptk signaling and Plcγ/IP_3_/Pkc/p38Mapk/Tnfα signaling, raising the question whether these are two coordinated events or mutually distinct responses.

### Validation of microarray data and differential expression of genes known to be affected by hyperoxaluria

**Figure 6:**
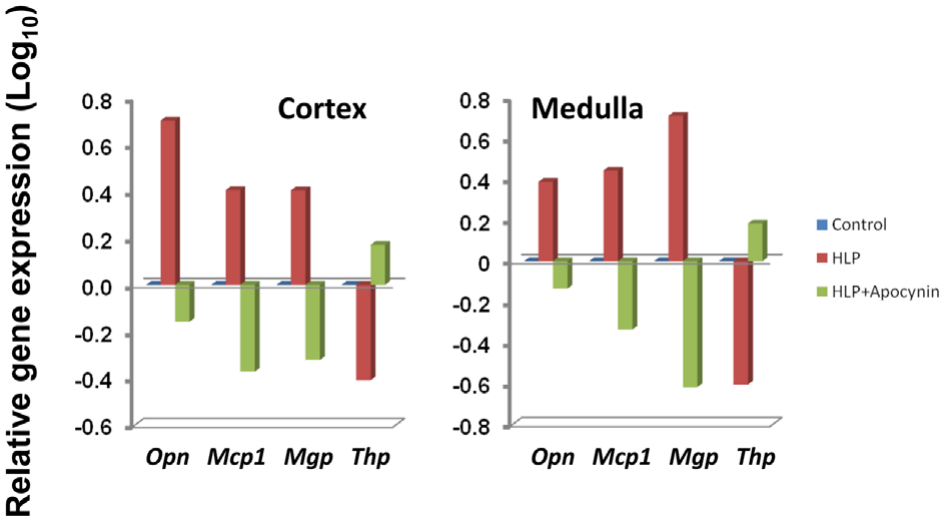
Relative expressions of selected genes encoding proteins in the HLP-fed rats and the HLP+Apocynin fed rats. As expected the HLP-fed rats had osteopontin (*Opn*), monocyte chemoattractant protein (*Mcp1*) and matrix gla protein (*Mgp*) to be up regulated and Tamm-horsefall protein (*Thp*) to be down regulated. The results were reversed with the HLP+Apocynin treated group. Most of the genes depicted had values >0.3 (or >2 fold ratio differential-expression).

**Figure 7:**
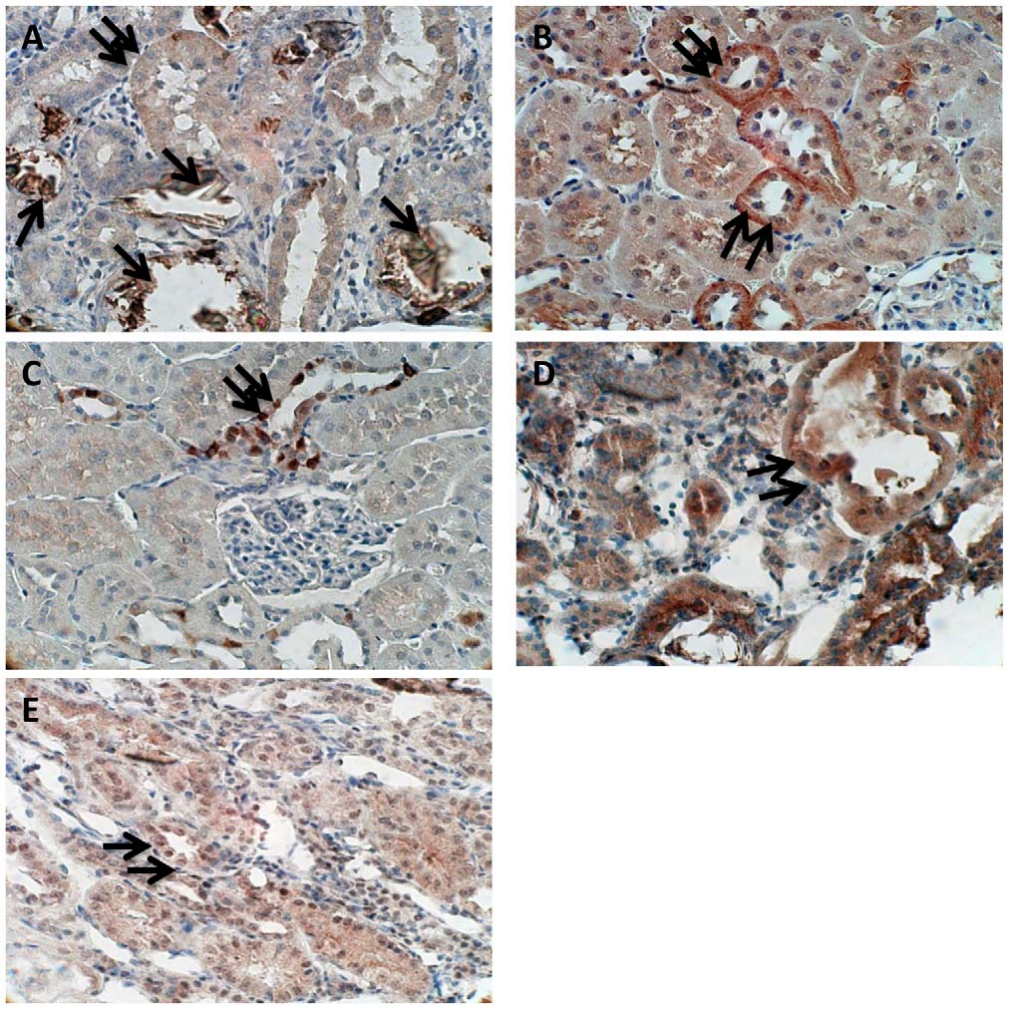
Immuno histochemical staining of paraffin embedded kidney sections of HLP treated rats. (**A**) Osteopontin (Opn) associated with crystal deposits, Single arrows show staining of crystal deposits while double arrows point to staining of epithelial cells of the adjoining renal tubular epithelia. (**B**) Double arrows point to strong staining for the monocyte chemoattractant protein (Mcp-1) in renal tubular epithelial cells (**C**) Staining for Nox-4 in tubular epithelial cells (**D**) Staining for Nox-2 in both tubular cells as well as interstitium and (**E**) general cytosolic staining for p47 in tubular epithelial cells.

Multiple published studies have clearly shown that a set of specific proteins are differentially-expressed in the kidneys of rats in response to treatments that induce a state of hyperoxaluria [30], [37]–[44]. These proteins include Osteopontin (encoded by *Opn*), Mcp-1 (encoded by *Ccl2*), and Matrix-Gla protein (encoded by *Mgp*) which tend to be up-regulated, and the inhibitor Tamm-Horsfall protein (encoded by *Thp*) that appears to be down-regulated [44]. In the current study, and presented in Figure 6, the genes for these four proteins are also differentially-expressed in both renal cortex and medulla tissues of HLP-fed rats, with *Opn, Ccl2* and *Mgp* each up-regulated and *Thp* down-regulated, as would be predicted from our previous results (28). This gene profile was reversed in HLP+Apocynin-fed rats. Immunostaining was then used to determine the protein expressions of Opn, Mcp-1, and both p47 and Nox2 subunits of NADPH oxidase in kidney sections. Opn, Mcp-1, and NADPH oxidase expressions were almost non-existent in the normal renal tubules, as well as those without crystal depositions, but unmistakable in renal tubules of the rats with heavy CaOx crystal depositions. Strong epithelial staining was observed in the crystal-associated sections of the tubules, but some crystal free tubules also revealed staining (Fig. 7). These representative data indicate a high correlation between transcriptome data and observed changes in protein expressions following HLP treatment.

## Discussion

Kyoto Encyclopedia of Genes and Genome (KEGG) pathway analysis of some 3000 genes per run (using a p-value <0.1 cut-off) was carried out for two different sets of genes, one cortex tissue-derived and the other medulla tissue-derived. In this way, we were able to compare the differential expression of renal tissue genes between rats exhibiting primarily hyperoxaluria/CaOx deposition and healthy non diseased rats. Using the pathway analysis of these 3000 gene sets by DAVID (Database for Annotation, Visualization of Integrated Discovery) enrichment analysis tool from National Institute of Allergy and Infectious Diseases (NIAID), we identified several pathways which were highly significant and unique within the experimental groups. Using four common genes whose proteins are well-documented to be differentially-expressed with development of calcium-oxalate urolithiasis (*Spp1, Ccl2, Mgp* and *Umod*), we were able to show that each gene exhibited an expected up-regulated or down-regulated expression corresponding to their reported protein expression.

Two of these gene products, Opn (encoded by *Spp1*) and Mcp-1 (encoded by *Ccl2*), are critical elements for the present study. Opn is an important protein component of stone matrix, thought to inhibit growth, nucleation and aggregation of calcium oxalate crystals under normal conditions [22], [45], [46]. Previous studies have shown that Opn expression in the kidneys was significantly increased due to hyperoxaluria and deposition of CaOx crystals [39]. Our results also showed an up-regulation of *Opn* transcripts in HLP fed rats. Opn is a ubiquitous protein associated with inflammation, cell proliferation and tissue remodeling after cell injury. Ox and CaOx crystals are toxic to renal epithelial cells [14], [16], [47], inducing the production of ROS-associated Opn which, in turn, modulates the adherence of CaOx crystals to renal epithelial cells. Not surprising, then, Opn is involved in activating a number of pathways, including focal adhesion maturation regulating cell-cell interactions and cell migrations. Mcp1/Ccl2, besides being a strong chemokine for monocytes and lymphocytes, is a general chemoattractant for inflammatory cells and an important factor in the renin/angiotensin/ROS pathway [43], [48]. The transcriptome data present herein support each of these roles.

Genes encoding proteins involved in the ROS pathway during development of hyperoxaluria and CaOx crystal deposition in the kidneys suggest two distinct molecular pathways that meld into a similar outcome. First, analyses of differentially-expressed genes from cortical and medullar sections of kidneys explanted from HLP-treated rats revealed similar activations of the multiple genes associated with assembly of NADPH oxidase complexes, as well as genes encoding upstream proteins indicating activation of the rennin/angiotensin/angiotensin receptor-2 signaling pathway(s). Interestingly, however, we also discovered the concomitant up-regulation of genes encoding both gp91*^Phox^* (Cybb or Nox2) and Nox4, two core proteins that define two distinct NADPH oxidase membrane complexes expressed on different tissues [49]. This latter finding has been further supported in our analyses of gene expressions in renal cortex and medulla tissues from kidneys extracted from rats exhibiting mostly hyperoxaluria versus hyperoxaluria with crystal depositions, where *gp91* and *Nox4* showed exclusive differential expressions (data not presented). Specifically, up-regulated *gp91* expression correlated in both the cortex and medulla tissues with CaOx crystal deposition, while *Nox4* expression best correlated in the medulla with hyperoxaluria. In any event, the presence of CaOx crystals in either renal cortex or medulla tissues was associated with a strong down-regulation of catalase (*Cat*) and superoxide dismutase (*Sod1*) gene expressions, two ROS scavenger enzymes, whose expressions are possibly determined by CaOx. We propose, therefore, that these preliminary data suggest at least two distinct molecular mechanisms are activated by hyperoxaluria and CaOx crystal depositions in the formation of renal NADPH oxidase complexes, but unraveling this will require further studies.

The fact that oxalate and CaOx crystals appear to induce strong ROS activation, especially in the absence of any concomitant up-regulation of ROS scavengers such as SOD and catalase, suggests the renal tissues during prolonged hyperoxaluria are bathed in free radicals and molecules with unpaired electrons, including superoxide anion (O_2_^–^), hydroxyl radical (OH), and hydrogen peroxide (H_2_O_2_). O_2_^−^ anions are produced by NADPH oxidases, xanthine oxidase, lipooxigenase, cyclooxygenase, hemeoxygenase, as well as a byproduct of mitochondrial respiration. While lipid radicals can also produce O_2_^−^, NO Radicals are produced by the endothelial nitric oxide synthase (eNOS) mediated oxidation of L-arginine. Not surprising, then, that the arginine/proline metabolism pathway is identified as an activated biological process by KEGG. In addition, reactions between superoxide and nitric oxide can produce the highly reactive peroxynitrite molecule, ONOO^−^. Since oxidative stress ultimately leads to injury and inflammation, it is not surprising that additional biological processes identified in KEGG analyses include cellular recruitment, various inflammatory responses, cellular stress, apoptosis and functional compensations, as listed in Tables 1 and 2. While the present study focused specifically on the NADPH oxidase/ROS system that involves a relatively few genes (or gene sets), the listing of signaling pathways defined by KEGG indicate that hyperoxaluria *per se* and blocking of NADPH oxidase assembly by Apocynin activates a myriad of similar and dissimilar biological processes that reveals a high level of complexity. Sorting out the biological processes defined by genes within each of these signaling pathways will require additional analyses, most likely global analysis. Perhaps the more interesting observation of the present study, however, has been the effects of Apocynin treatment *per se* on the gene expression profiles in rat renal tissues induced *in vivo* by HLP, specifically, the full reversal of these up-regulated genes in renal tissues of HLP+Apocynin-fed rats irrespective of having no effect on reducing hyperoxaluria [30]. This observation is even more interesting if one considers the observed effects are seen at the level of transcription. How Apocynin affects gene transcription will also require further studies.

**Table 2:** Functional annotation chart showing differentially-expressed pathways in renal medulla tissue comparing HLP+ Apocynin treated versus untreated control groups.

Category	Signaling Pathway	Count	Percentage	p-value	Benjamini
**KEGG_PATHWAY**	ErbB signaling pathway	14	1.9	1.5E-5	4.1E-2
**KEGG_PATHWAY**	TGF-beta signaling pathway	14	1.9	1.7E-3	3.5E-2
**KEGG_PATHWAY**	MAPK signaling pathway	28	3.7	4.7E-3	7.5E-2
**KEGG_PATHWAY**	Chemokine signaling pathway	20	2.7	6.6E-3	8.7E-2
**KEGG_PATHWAY**	Non-small cell lung cancer	9	1.2	1.2E-2	1.1E-1
**KEGG_PATHWAY**	Insulin signaling pathway	16	2.1	1.2E-2	1.1E-1
**KEGG_PATHWAY**	Focal adhesion	21	2.8	1.3E-2	1.1E-1
**KEGG_PATHWAY**	Complement and coagulation cascades	10	1.3	2.3E-2	1.7E-1
**KEGG_PATHWAY**	Gap junction	10	1.3	5.3E-2	2.7E-1
**KEGG_PATHWAY**	Fc epsilon RI signaling pathway	9	1.2	8.4E-2	3.7E-1
**KEGG_PATHWAY**	Chronic myeloid leukemia	17	2.3	6.7E-6	1.1E-3
**KEGG_PATHWAY**	Pathways in cancer	36	4.8	2.8E-4	2.3E-2
**KEGG_PATHWAY**	Cell cycle	19	2.5	4.7E-4	2.6E-2
**KEGG_PATHWAY**	Pancreatic cancer	13	1.7	7.2E-4	2.9E-2
**KEGG_PATHWAY**	Neutrophin signaling pathway	18	2.4	1.3E-3	4.2E-2
**KEGG_PATHWAY**	Lysosome	17	2.3	1.6E-2	3.6E-2
**KEGG_PATHWAY**	Sphingolipid metabolism	9	1.2	3.1E-3	5.5E-2
**KEGG_PATHWAY**	Acute myeloid leukemia	10	1.3	5.0E-3	7.3E-2
**KEGG_PATHWAY**	Renal cell carcinoma	11	1.5	7.6E-3	9.3E-2
**KEGG_PATHWAY**	Colorectal cancer	12	1.6	8.6E-3	9.7E-2
**KEGG_PATHWAY**	Small cell lung cancer	12	1.6	1.0E-2	1.1E-1
**KEGG_PATHWAY**	Fc gamma R-mediated phagocytosis	12	1.6	1.6E-2	1.3E-1
**KEGG_PATHWAY**	Prion disease	7	0.9	1.7E-2	1.3E-1
**KEGG_PATHWAY**	Notch signaling pathway	8	1.1	2.6E-2	1.8E-1
**KEGG_PATHWAY**	Glioma	9	1.2	2.8E-2	1.9E-1
**KEGG_PATHWAY**	Endometrial cancer	8	1.1	3.1E-2	2.0E-1
**KEGG_PATHWAY**	Endocytosis	20	2.7	3.2E-2	2.0E-1
**KEGG_PATHWAY**	P53 signaling pathway	9	1.2	4.3E-2	2.4E-1
**KEGG_PATHWAY**	Glycerolipid metabolism	7	0.9	4.6E-2	2.5E-1
**KEGG_PATHWAY**	T-cell receptor signaling pathway	12	1.6	6.2E-2	3.1E-1
**KEGG_PATHWAY**	Bladder cancer	6	0.8	6.2E-2	3.0E-1
**KEGG_PATHWAY**	Apoptosis	10	1.3	6.8E-2	3.1E-1
**KEGG_PATHWAY**	Prostate cancer	10	1.3	9.0E-2	3.8E-1
**KEGG_PATHWAY**	Toll-like receptor signaling pathway	10	1.3	9.0E-2	3.8E-1
**KEGG_PATHWAY**	mTOR signaling pathway	7	0.9	9.6E-2	3.9E-1

The gene heading indicates number of genes mapped to an ontology category. The first ten pathways are common in the cortex and the medulla. P-value derived from Fisher's exact test and Benjamini multiple test correlation.

Previous research has suggested that, based on the core protein(s) of Flavocytochrome b_558_, seven distinct isoforms of NADPH oxidase exist: Nox1 to Nox 5 and the structurally similar Duox1 and Duox2 molecules [50]. These various isoforms have different functions, e.g., Nox2 leads to respiratory burst of leukocytes and is involved in innate immunity, while Nox3 is needed for balance sensing and formation of otoliths. Furthermore, various isoforms are expressed in different cell types, e.g., Nox1 is expressed primarily in smooth muscle cells [51], with little or no expression in endothelial cells [52], while Nox2 has been reported to be expressed in endothelial cells [53] and adventitious fibroblasts [54]. On the other hand, Nox4, while strongly expressed in endothelial cells [55], is also found in both adventitial fibroblasts [54] and smooth muscle cells [56]. Studies have also shown that both Nox1 and Nox2 are expressed in plasma membranes, endosomes and caveolae [57], [58], while Nox4 has been shown to be localized in focal adhesions, the endoplasmic reticulum and the nucleus [58]–[61].

Considering the complex structure of the kidney, it is not surprising that there is also a wide distribution in various NADPH oxidase subunit expressions, including the multiple isoforms of Nox. ROS are produced by various cell types of the kidneys, such as tubular cells, endothelial cells, vascular smooth muscle cells, fibroblasts and podocytes [62]. Similarly, Nox isoforms, such as Nox4 and Nox1, along with other subunits of the NADPH oxidase complex, are known to be expressed in distal tubules, collecting ducts, glomeruli, renal vessels and macula densa [62], [63]. Previous research has shown that Nox1 is predominantly expressed in the colon [51], [64], Nox3 in the fetal kidney [65], Nox4 in the renal cortex and Nox5 in the spleen, with Duox1 and Duox2 restricted to the thyroid [62]. Nox 4 has also been shown to be the predominant Nox isoform widely distributed in the kidney in vasculature, glomeruli, mesangial cells and nephron segments [66], [67]. In line with these observations, the present study indicates that expressions of *Nox4* and *Nox1* are differentially-expressed in renal tissue during development of hyperoxaluria, whereas we did not find differential expression of *Nox3, Nox5, Duox1* and *Duox2* (data not shown).

Lastly, one cautionary note must be considered in interpretation of the current data. Although analyses of transcriptome data are a powerful tool, it is also dependent on a number of variables, one of which is the starting tissue. In the current study, it must be pointed out that dissection of the kidney into medulla and cortex is technically difficult, leading to the strong probability that each may have some contaminating cells derived from the other tissue fraction. Similarly, there was no attempt in the current study to isolate renal tissue from infiltrating neutrophils, a fact that no doubt affects the transcriptome data. Thus, assigning precise molecular mechanisms to specific tissues or regions will require more detailed studies. Nevertheless, an in-depth analysis of the genes that identify these specific biological pathways or cellular compartments should provide important up-stream and down-stream steps in renal tissue responses to hyperoxaluria, subsequent development of CaOx crystals and CaOx crystal depositions leading to an eventual onset of the inflammatory responses which clearly involve ROS production. Of clear importance, the current study provides strong evidence that multiple unique and similar changes are occurring in the renal tissues at the molecular and cellular level, whether the rats are treated with HLP or with HLP+Apocynin, despite minimal changes noted at the gross level. These observations raise the question whether apocynin is merely an inhibitor of NADPH oxidase complex assembly or a molecule with strong anti-oxidant inductive activities. Further analyses of the extensive transcriptome data may provide additional clues.

## Supporting Information

Figure S1 **Volcano plot of the comparison between HLP treated Vs Control for cortex and medulla.** Each point represents a gene. The x-axis represents the Log2 transformed fold changes and the y-axis represents the log10 transformed p-values. In our case, a log2 value of 1 means that the average gene expression level in group 2 has a two-fold positive change compared to that in the group 1. Comparison results having a p-value <0.01 is highlighted in red.
(TIF)
