# Supplementary figures and images for: Apocynin-Treatment Reverses Hyperoxaluria Induced Changes in NADPH Oxidase System Expression in Rat Kidneys: A Transcriptional Study
Source: PLoS One. 2012 Oct 16;7(10):e47738. doi: 10.1371/journal.pone.0047738 (PMC3473023; doi:10.1371/journal.pone.0047738)

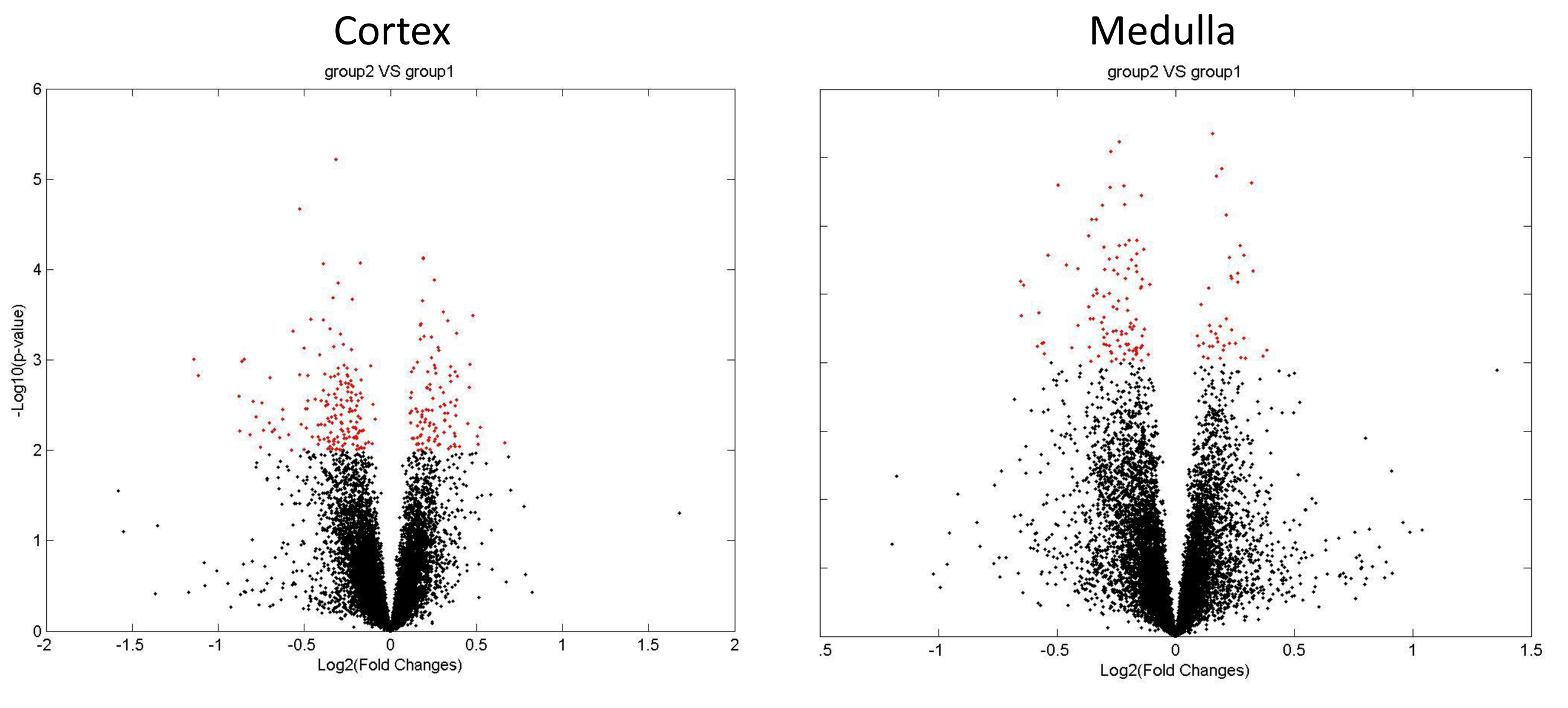

Supplement: Figure S1 — Volcano plot of the comparison between HLP treated Vs Control for cortex and medulla. Each point represents a gene. The x-axis represents the Log2 transformed fold changes and the y-axis represents the log10 transformed p-values. In our case, a log2 value of 1 means that the average gene expression level in group 2 has a two-fold positive change compared to that in the group 1. Comparison results having a p-value <0.01 is highlighted in red. (TIF) [file pone.0047738.s001.tif]
